# Supplementary material for: Independent representations of ipsilateral and contralateral limbs in primary motor cortex
Source: eLife. 2019 Oct 18;8:e48190. doi: 10.7554/eLife.48190 (PMC6824843; doi:10.7554/eLife.48190)
Supplement: Supplementary file 1. — Significance assessed based on sampling from a uniform distribution. *p<0.05, **p<0.01, ***p<0.001 [file elife-48190-supp1.docx]

**Supplementary File 1: Comparison of Rayleigh statistic between the non-overlapping subset of neurons and the original neuron population. Significance assessed based on sampling from a uniform distribution. * p<0.05, **p<0.01, ***p<0.001**

| **Rayleigh R-Statistic between tuning distributions** | **Non-Overlapping Neurons** | **Original Neuron Population** |
| --- | --- | --- |
| Pert Epoch: Contra Tuning – Ipsi Tuning | Monkey P: R= 0.09  Monkey M: R=0.23* | Monkey P: R=0.14  Monkey M: R=0.24** |
| Steady-State Epoch: Contra Tuning – Ipsi Tuning | Monkey P: R=0.18  Monkey M: R=0.18 | Monkey P: R=0.14  Monkey M: R=0.24** |
| Contra Loads: Perturbation Tuning – Steady-State Tuning | Monkey P: R=0.63***  Monkey M: R=0.57*** | Monkey P: R=0.65***  Monkey M: R=0.57*** |
| Ipsi Loads: Pert Tuning – Steady-State Tuning | Monkey P: R=0.55***  Monkey M: R=0.44*** | Monkey P: R=0.5***  Monkey M: R=0.44*** |
