# Supplementary material for: Independent representations of ipsilateral and contralateral limbs in primary motor cortex
Source: eLife. 2019 Oct 18;8:e48190. doi: 10.7554/eLife.48190 (PMC6824843; doi:10.7554/eLife.48190)
Supplement: Supplementary file 2. [file elife-48190-supp2.docx]

**Supplementary File 2: Comparison of firing magnitude between the non-overlapping subset of neurons and the original neuron population**

| **Magnitude** | **Non-Overlapping Neurons**  **(Median, IQR)** | **Original Neuron Population**  **(Median, IQR)** |
| --- | --- | --- |
| Pert Epoch:  Contralateral (Hz/Nm) | Monkey P: 43(22.8, 59.9)  Monkey M:23.4 (10.3, 38.3) | Monkey P: 39.2(19.7, 56.4)  Monkey M:24.3(10.7, 37.9) |
| Pert Epoch: Ipsilateral (Hz/Nm) | Monkey P: 16.4(7.2, 35.6)  Monkey M:13 (7.6, 26.7) | Monkey P: 16.4 (7.3, 32.4)  Monkey M:13.4(8.1, 26.3) |
| Steady-State Epoch: Contralateral (Hz/Nm) | Monkey P:26.3(15.5, 40.7)  Monkey M: 14.4(7.6, 26.1) | Monkey P: 24.5(13.6, 38.1)  Monkey M: 15.6(8.7, 25.9) |
| Steady-State Epoch: Ipsilateral (Hz/Nm) | Monkey P: 10(5.4, 15.8)  Monkey M: 9.7(6, 15.6) | Monkey P: 10(5.8, 15.4)  Monkey M: 9.7(5.4, 16.2) |
