# Supplementary material for: Independent representations of ipsilateral and contralateral limbs in primary motor cortex
Source: eLife. 2019 Oct 18;8:e48190. doi: 10.7554/eLife.48190 (PMC6824843; doi:10.7554/eLife.48190)
Supplement: Supplementary file 3. [file elife-48190-supp3.docx]

**Supplementary File 3: Comparison of onset timing between the non-overlapping subset of neurons and the original neuron population**

| **Onset of Load-Related Activity** | **Non-Overlapping Neurons**  **(Median, IQR)** | **Original Neuron Population**  **(Median, IQR)** |
| --- | --- | --- |
| Contralateral Onset (ms) | Monkey P: 60(37, 26.5)  Monkey M:76 (64.5, 86.5) | Monkey P: 64.5(41.5, 83.5)  Monkey M:76 (68.2, 93.3) |
| Ipsilateral Onset (ms) | Monkey P:82(57.8, 104.8)  Monkey M: 105 (77, 158) | Monkey P:78.5(59, 106)  Monkey M: 117 (77, 154) |
